# Genetic parameters of growth and leaf traits and genetic gains with MGIDI in three *Populus simonii* × *P*. *nigra* families at two spacings

**DOI:** 10.3389/fpls.2024.1483580

**Published:** 2024-12-24

**Authors:** Tianxin Wang, Jingshan Ren, Qinjun Huang, Jinhua Li

**Affiliations:** Research Institute of Forestry, Chinese Academy of Forestry, Beijing, China

**Keywords:** *P. simonii* × *P. nigra*, genetic parameters, genotype and environment interaction (GEI), MGIDI, leaf morphology and anatomy

## Abstract

New genotypes of hybrid from the *Aigeiros* and *Tacamahaca* sections, which encompass economically important species of *Populus* L., have great potential to significantly enhance genetic gain from selection. Growth and its functional and structural determinants exhibiting a high level of variability are not only controlled by genetics, but also affected by environment, as well as genotype and environment interaction (G×E). The preceding research on the intersectional progenies derived from eight families (*P*. *simonii* × *P*. *nigra*) and their respective parents has indicated that leaf traits exhibiting robust genetic control were employed for selection of hybrid genotypes displaying multiple traits. The goals of this research with the progenies 3 families across two spacing trials were to (1) assess the GEI in progeny genotypes for multiple traits, (2) estimate the genetic parameters for important traits, (3) identify the genotypes with superior productive performance, adaptability, and genotypic stability using the MGIDI index, (4) select genotypes that exhibit high performance and genotypic stability across multiple traits using the MGIDI index. We found that the progeny genotypes showed considerable variation in growth and leaf morphology response to the spacings and genotype interaction effects were significant (P ≤ 0.001) for most of the traits studied in the progeny of each family and the joint family. The highest broad-sense heritability was observed for petiole length, while the lowest heritability values were recorded for stomatal length among the eight traits studied in both each family and the joint family. The MGIDI, assuming selection intensity of 15%, identified 26, 25, 35, and 86 genotypes in the three families and the joint family, respectively. The selected hybrids of each family and the joint family exhibited the desired genetic gains, including positive gains for leaf area (6.87%-11.2%), petiole length (3.81%-13.7%) and plant height (1.30%-10.4%). The interpretation of strengths and weaknesses as illustrated by the MGIDI provides guidance for the breeders to develop poplar hybrids performed well in desired traits, such as growth and other yield contributors i.e. leaf traits. The tested progeny genotypes of three families provided a valuable addition to the hybrid selection for rapid juvenile growth.

## 1 Introduction

*Populus* L. is a genus native to the northern hemisphere, with the capacity to grow in a wide range of climatic and soil conditions. As one of the fastest-growing trees in temperate latitudes, poplars are of considerable commercial importance in many regions of the world (Dickmann and Kuzovkina, 2014; Zsuffa et al., 1996). Providing approximately 20 million m^3^ of wood and fiber annually, poplars are desirable due to their rapid growth and versatile use of the wood (FAO, 2021; Kutsokon et al., 2015). In addition to a wide range of wood products (such as industrial round wood and poles, pulp and paper, reconstituted panels, plywood, veneer, sawn timber, packing cases, pallets and furniture), as well as non-wood products (such as fodder, fuelwood), poplars provide many important environmental functions such as restoring degraded land, safeguarding soils and water, preserving biological diversity, providing shelter and shade, and sequestering carbon in temperate climates (Gardiner et al., 2024; He et al., 2018; Kutsokon et al., 2015; Isebrands et al., 2014; Singh and Kumar, 2012; Sigaud, 2011). It is estimated that the total area of poplars is 59 million hectares, well presented in China, Canada, Turkey and the USA. However, planted poplars represent a small component of landscape strategy for efficient production of ecosystem goods and services (FAO, 2021; Kutsokon et al., 2015). Poplar plantations cover a total area of 8 million acres, which 61% is used for industrial roundwood, 23% for environmental protection, 9% for fuelwood biomass and 7% for other purposes. There is increasing interest in the selection and improvement of poplars for fast-growing, high-yielding SRC plantations, which have the potential to become sustainable sources of significant supply. To sustainably increase the productivity of genotypic material and produce an appropriate level of genetic variation in the genetic materials that are commercially available, ongoing breeding and selection operations are necessary for the productivity of poplar plantation (Adler et al., 2021; Yáñez et al., 2019; Verlinden et al., 2013; Sixto et al., 2011; Pellis et al., 2004).

The *Populus* genus is notable for its considerable diversity and variability, manifesting in numerous aspects including morphology, biomass production, and resistance to biotic and abiotic stresses (Biselli et al., 2022; Stettler et al., 1996). Of the six taxonomically distinct sections of the genus (Dickmann and Kuzovkina, 2014; Eckenwalder, 1996), the sections *Aigeiros* and *Tacamahaca* comprise the majority of economically important species, which are sexually compatible and occur naturally interspecific hybridization (Stettler et al., 1996). Interspecific and intersectional hybrids have been observed to exhibit enhanced growth vigour, superior adaptability, fecundity, stress resistance and other traits in comparison with their parental species, which achieved remarkable economic benefits (Ren et al., 2020; Rodríguez et al., 2019; Marron et al., 2006; Bisoffi and Gullberg, 1996). Significant discrepancies in productivity and in its functional and structural determinants have been documented among poplars, particularly within the sections *Aigeiros* and *Tacamahaca* and their hybrids (Gebauer et al., 2016; Verlinden et al., 2013; Al Afas et al., 2007; Marron and Ceulemans, 2006; Rae et al., 2004; Li et al., 2002). The relevance of various traits, both at the whole plant and at the leaf levels, as determinants of productivity has already been the subject of study in the context of poplar. A productivity determinant can be defined as a characteristic that is associated with the observed differences in productivity levels between trees, as a result, may serve as a potential indicator of this productivity level (Guet et al., 2015; Verlinden et al., 2013; Al Afas et al., 2007; Pellis et al., 2004; Ridge et al., 1986). From a practical standpoint of view, breeders are interested in early and easily measurable indicators of the future performance of the poplar genotypes with plantation purposes. A number studies have demonstrated that leaf morphological characteristics are indicative of long-term clonal performance and growth (Marron and Ceulemans, 2006; Marron et al., 2005; Harrington et al., 1997). Stomatal traits have been extensively investigated in *Populus*, as these traits are a valuable criterion for production and clone discrimination in the genus (Russo et al., 2014; Al Afas et al., 2007; 2006; Orlovic et al., 1998). For example, the effect of stomatal density on biomass production has been demonstrated in different poplar clones (Čortan et al., 2017; Russo et al., 2014; Al Afas et al., 2006). The characteristics of the stomata are highly dependent on the genetic background of the plants, as well as on the conditions of their growth and the stage of their development (Renninger et al., 2021; Čortan et al., 2017; Al Afas et al., 2006). A considerable number of studies attempted to establish a correlation between the variability in productivity and tolerance to environmental changes and the variability in leaf characteristics. However, the success of these studies has varied depending on the growth conditions (Renninger et al., 2021; Zhang et al., 2020; Niemczyk et al., 2019; Bonhomme et al., 2008). The indirect selection efficiency of these traits is primarily dependent on their genetic correlation with the target objective, as well as on the higher heritability values observed under the relevant testing conditions (Niemczyk and Thomas, 2020; Verlinden et al., 2013; Marron et al., 2007; Al Afas et al., 2007). Consequently, the question of whether stable determinants can be identified remains unanswered. Furthermore, only a limited number of studies have investigated the relevance of leaf morphology and stomatal characteristics for this purpose.

The variability observed in growth and associated biomass production is not solely attributable to genetics (Drost et al., 2015; Marron and Ceulemans, 2006), but is also influenced by environmental factors, as well as the interaction between genotype and environment (G×E) (Li et al., 2021; Li et al., 2017; Sixto et al., 2011; Zalesny et al., 2009). Among the factors affecting growth and associated biomass production, stand density and site nutritional status are of particular important for the optimal productivity of plantations (Toillon et al., 2021; Mamashita et al., 2015). The morphological characteristics of the poplar genotypes or clones were identified in order to ascertain the main characteristics that result in superior growth under different environmental conditions. The leaf provides an important perspective for understanding how plants respond and adapt to environmental changes (Niklas et al., 2023; Liu et al., 2023; 2019; Li and Wang, 2021). It has been demonstrated that different genetic entries might exhibit variations in growth and leaf morphology in response to distinct management regimes and different environmental conditions (Tsarev et al., 2021; Mamashita et al., 2015; Benomar et al., 2012; Dillen et al., 2007; Zalesny et al., 2009; Bonhomme et al., 2008). The current understanding of the growth and morphological responses of *Populus* genotypes and clones when growing under different environment is incomplete. Genotype by environment interactions (G × E) have been documented in numerous trials of *P*. *deltoides* and *P*. *euramericana* in China and the United States (Zalesny et al., 2009; Nelson et al., 2018; Liu et al., 2021; 2023; Li, 2021; Yuan et al., 2022). The majority of existing research has concentrated on the genetic by environment interaction (G×E) for growth traits across different sites. A limited number of studies have examined the genotypic and spacing effects on growth traits and leaf attributes of poplar, utilizing a small number of clones or varieties (Gardiner et al., 2024; Ghezehei et al., 2021; Zhang et al., 2020; Ridge et al., 1986). There is a paucity of studies that have examined genotypic variation among different poplar genotypes at different family or population levels for a wide range of different parameters. For example, Otis-Prud’homme et al. (2023); Gudynaitė-Franckevičienė and Pliūra (2022); Ding et al. (2020); Zhang et al. (2020) and Pliura et al. (2014)) have contributed to this field of research. However, there is still a lack of understanding regarding the joint influence of genetics, spacing and their interaction on poplar growth and associated production traits. This is particularly important for optimally breeding and managing of poplar plantations.

More recently, phenotypic selection integrating multi-environment data has emerged in the forest breeding community in order to increase accuracy by modelling G×E rather than ignoring it (Alexandru et al., 2023; Alves et al., 2018; Li et al., 2017). In the poplar-breeding programs, genetic evaluation and selection of super individuals through multi-environment trials (MET) is necessary and refers to changes in genotype ranking across environments (Liu et al., 2023; Yuan et al., 2022; Liu et al., 2021; Li, 2021; Jiang et al., 2021; Álvarez et al., 2020). Due to environmental variation, phenotype variation also occurs as a function of G×E, which is one of the major problems of breeding programs of any species, whether in the stages of selection or variety recommendation (Adler et al., 2021; Gudynaitė-Franckevičienė and Pliūra, 2021; Sixto et al., 2011). In this context, the mixed model method is considered as more accurate (Piepho et al., 2008; Yang, 2007), as it provides better experimental precision and is more efficient than analysis of variance, especially in cases with unbalanced data. Mixed models are used as an optimal selection procedure and include the estimation of variance components through the restricted maximum likelihood model (REML) and the prediction of genotypic values by the best linear unbiased prediction (BLUP), resulting in a selection with higher accuracy (Yuan et al., 2022; Liu et al., 2021; Li, 2021; Alves et al., 2018). Furthermore, the predicted genetic values can be used to estimate the adaptability and stability of genotypes using the harmonic mean of the relative performance of genetic values (HMRPGV), which allows simultaneous estimating adaptability and stability in a single parameter (Vidal et al., 2022). The utilization of selection represents a further avenue for enhancing the efficacy of the process, which is conducted on multiple traits concurrently, yielding genotypes with superior performance and closer to the ideotype (Rocha et al., 2018). In a recent study, Olivoto and Nardino (2021) compared several multi-trait selection indices and proposed the novel multi-trait genotype–ideotype distance index (MGIDI) for the selection of genotypes based on breeding values, with consideration of information from multiple traits (Olivoto et al., 2022; Olivoto and Nardino, 2021; Olivoto et al., 2021). In contrast to conventional ranking methods utilizing *post-hoc* tests, such as Fisher’s least significant differences (LSD) or Tukey’s honest significant difference (HSD) in ANOVA, which permit ranking for a single test only, the MGIDI index enables the ranking of genotypes based on their performance across multiple traits. Moreover, the MGIDI circumvents issues of collinearity by employing factor analysis for indexing (Olivoto and Nardino, 2021; Olivoto et al., 2019a; b). This technique has been successfully employed in a number of crop breeding programs, including those focused on wheat (Pour-Aboughadareh and Poczai, 2021), rice (Jalalifar et al., 2023), maize (Singamsetti et al., 2023), water yam (Ouattara et al., 2024), sweet potato (Alam et al., 2024), etc. Two multi-trait selection indices (MGIDI and MTSI) have been employed in order to combine growth with quality traits and survival rate in order to select the most valuable and stable Norway spruce provenances (Alexandru et al., 2023).

The preceding research on the leaf size traits of the intersectional progenies from eight families (*P*. *simonii* × *P*. *nigra*) and their parents has documented (Ren et al., 2020). This research indicated that leaf morphology traits, which are subject to strong genetic control, were employed for the selection of hybrid genotypes and crossing combinations based on the breeding values of multiple traits. The objective of the research was to identify promising hybrids based on a combination of traits, including growth, leaf stomata and morphological characteristics under different spacing trials. The present study employed three of the eight families with the following objectives: (1) To assess the GEI in progeny genotypes for multiple traits, (2) To estimate the genetic parameters for important traits, (3) To identify the genotypes with superior productive performance and genotypic stability using the MGDI index.

## 2 Materials and methods

### 2.1 Plant material

The experimental material comprises three of eight families resulting from the intersectional crosses between three female clones of *P*. *simonii* (‘1-XY’, ‘XY-5’) and *P*. *pseudo*-*simonigra* (‘ZL-3’) from China, and six male clones of *P*. *nigra* (‘N188’, ‘N020’, ‘N139’, ‘N151’, ‘N429’, ‘N430’) from Italy (Wang et al., 2020; Ren et al., 2020). All of the crosses were conducted at the Chinese Academy of forestry (CAF) in Beijing in 2014 and were repeated at the Tangshan Base in Hebei Province in 2017 to increase the size of the progeny. The three selected families were the subject of investigation in this study and were included in F_1_ hybrid genotypes resulting from three interspecific crosses: *P*. *simonii* ‘1-XY’×*P*. *nigra* ‘N139’ (‘1-XY’×‘N139’), *P*. *simonii* ‘1-XY’×*P*. *nigra* ‘N188’ (‘1-XY’×‘N188’) and *P*. *pseudo*-*simonigra* ‘ZL-3’×*P*. *nigra* ‘N188’ (‘ZL-3’×‘N188’).

### 2.2 Site description

The experimental trials were conducted at the Tongzhou Base of Research Institute of Forestry (RIF) in Beijing, Northern China (39°43’ N, 116°44’ E) at an elevation of approximately 27.6 meters above sea level. The climate is classified as continental, exhibiting notable fluctuations in temperature (with an annual mean of 13.8°C and extremes of -5.1°C in January and 25.7°C in July). The annual precipitation was 620.9 mm on average, with a pronounced maximum in the summer months (65% in July and August). The annual frost-free period was approximately 190 days. The soil is classified as sandy-loam, with a pH value of 7.13.

### 2.3 Experimental design

Two field trials with different plant spacing were established in May 2021 at the Tongzhou Base of RIF in Beijing, Northern China according to the randomized block design. In each trial, three complete blocks were defined, with each block comprising three replicates of each F_1_ genotype and each parent clone. The first spacing trial was 0.25 m × 1 m (E1), followed by the second trial with a spacing of 0.50 m × 1 m (E2). Both trials were planted with 25 cm homogeneous cuttings derived from 2-year-old trees in April, 2021. The plantation management included in irrigation once a month and the application of ground cloth for the purpose of weed removal from June to September in 2021.

### 2.4 Plant measurements

#### 2.4.1 Plant growth

Plant height (H, m) were measured in late September and early October 2021 after the growing season was finished. Each F_1_ progeny genotype of three families and the parental clone was represented and survived for the investigation by a minimum of two plants to a maximum of six plants.

#### 2.4.2 Leaf stomata and morphological characteristics

In September 2021, one average shoot per replicate plot was randomly selected as the sample plant for each progeny genotype and parental clone. One recently mature leaf was collected from the sixth to the ninth leaf on the upper top of each sample plant, then put into plastic bags with moistened filter paper and brought to the laboratory for leaf stomatal impressions and morphological traits. Petiole was cut from the leaf base and petiole length (PL, cm) was measured with a straightedge. All leaves from sample plants were imaged with the HP ScanJetG4010 scanner and saved as JPG (Joint Photographic Experts Group) files. Individual leaf area (LA, cm^2^), circumference (LC, cm), length (LL, cm) and width (LW, cm) were measured for all leaves with Digimizer software. Replicate impressions of abaxial leaf epidermis were taken at the point of maximum leaf width near the center vein of the leaf, using colorless nail polish (Al Afas et al., 2006). All stomatal impressions from leaf surfaces were fixed on glass slides and imaged under a light microscope (OLYMPUS BX51, with a camera device and projected on a screen) at a magnification of 40× objective lens, 10× eyepiece. Stomata were counted and stomatal density (SD) was calculated as the number of stomata per unit of leaf area. Stomatal length (SL, μm) was measured with Digimizer (MedCalc Software bvba) software (Wang et al., 2020).

### 2.5 Data analysis and software

The combined data from two spacing trial environments was conducted the individual analysis of variance and ensured the homogeneity of residual variances using the Shapiro-Wilk test, then the joint analysis of variance was performed. All data were analysis and formulated according to Singamsetti et al. (2023); Olivoto and Lúcio (2020).

#### 2.5.1 Variance component analysis

For two spacing trial environments, the traits were initially fitted into a linear mixed-effect model by considering genotype, genotype-by-environment, and incomplete blocks within complete replicates as random effect and spacings and complete replicates as fixed effect (Olivoto et al., 2019a). The following standard linear mixed model (Yang, 2007) was computed with the function *gamem_met*() from the metan package (Olivoto and Lúcio, 2020).


y=Xβ+Zu+ϵ

where *y* is a vector of response variable (such as height), *β* is a vector of fixed effects, u is a vector of random effects; *X* is a design matrix of 0s and 1s relating *y* to *β*, *Z* is a design matrix of 0s and 1s relating *y* to *u*, and *ϵ* is a vector of random errors.

The estimates of variance components were obtained by Restricted Maximum Likelihood (REML) using the expectation-maximum algorithm (Dempster et al., 1977). A likelihood ratio test (LRT) with a two-tailed chi-square test with one degree of freedom was performed to test the significance of the random effects. Broad-sense heritability ( $h_{g}^{2}$ ), was calculated as


$$h_{g}^{2}=\frac{\sigma_{g}^{2}}{\sigma_{g}^{2}+\sigma_{i}^{2}+\sigma_{e}^{2}}$$

where $\sigma_{g}^{2}$ is the where is the genotypic variance, $\sigma_{i}^{2}$ is the variance of the GEI interaction, and $\sigma_{e}^{2}$ is the residual variance. GEIr2 is the coefficient of determination of the interaction effects, $r_{i}^{2}$ , was estimated by


$$r_{i}^{2}=\frac{{\hat{\sigma }}_{i}^{2}}{\left[{\hat{\sigma }}_{g}^{2}+{\hat{\sigma }}_{i}^{2}+{\hat{\sigma }}_{e}^{2}\right]}$$

Heribatility of means is the heritability on the mean basis, $h_{mg}^{2}$ , was calculates as


$$h_{mg}^{2}=\frac{{\hat{\sigma }}_{g}^{2}}{\left[{\hat{\sigma }}_{g}^{2}+\frac{{\hat{\sigma }}_{i}^{2}}{e}+\frac{{\hat{\sigma }}_{e}^{2}}{eb}\right]}$$

Where ${\hat{\sigma }}_{g}^{2}$ , ${\hat{\sigma }}_{i}^{2},\;$ and ${\hat{\sigma }}_{e}^{2}$ are the variances related to genotypes, genotype–environment interaction, and error terms, respectively; *e* and *b* are the number of environments and replicate blocks per spacing trial environment, respectively. Accuracy is the accuracy of selection, $A_{c}$ , was calculated by


$$A_{c}=\sqrt{h_{mg}^{2}}$$

rge is the genotype-environment correlation, $r_{ge}$ , estimated by


$$r_{ge}=\frac{\sigma_{g}^{2}}{\sigma_{g}^{2}+\sigma_{i}^{2}}$$

CVg and CVr were the genotypic coefficient of variation and the residual coefficient of variation estimated, respectively, by


$$CV_{g}=\left(\frac{\sqrt{{\hat{\sigma }}_{g}^{2}}}{\mu }\right)\times 100$$

and


$$CV_{r}=\left(\frac{\sqrt{{\hat{\sigma }}_{e}^{2}}}{\mu }\right)\times 100$$

where μ is the grand mean. CV ratio is the ratio between genotypic and residual coefficient of variation.

#### 2.5.2 Genetic correlations

To better understand the inheritable relationships between eight traits studied and to see if these relationships are changed in the means and BLUPs across the spacing trial environments, a genetic correlation was performed on the means and BLUPs. The correlation matrix was represented as a network plot.

#### 2.5.3 The multi-trait genotype–ideotype distance index

The estimation of MGIDI values for test progenies in each spacing trial environment was based on two-way Best Linear Unbiased Predictions (BLUPs) for each genotype (row) and trait (column) and was carried out in four steps, i.e., rescaling of the studied traits, exploratory factor analysis (EFA) to reduce the dimensionality, planning for ideotype with maximum rescaled value, and calculation of Euclidean distance between the genotypes and ideotype planned as the MGIDI index (Olivoto and Nardino, 2021). Rescaling the traits was performed so that all have a similar range, i.e., 0–100. The rescaled value (*r*X*_ij_*) of the *j*th trait (column) of the *i*th genotype (row) was calculated using the following formula:


$$rX_{ij}=\frac{\eta_{nj}-\varphi_{nj}}{\eta_{0j}-\varphi_{0j}}\times \left(\theta_{ij}-\eta_{oj}\right)+\eta_{nj}$$

where *η*_0_ *_j_* and *φ*_0_ *_j_* are the original maximum and minimum values for the trait *j*, respectively; *θ_ij_* is the original value for the *j*th trait of the *i*th genotype/hybrid; and *η_nj_* and *φ_nj_* are the new maximum and minimum values for the trait *j* after rescaling, respectively. The values for *η_nj_* and *φ_nj_* are chosen according to the desirability as follows. For the 8 traits studied, H, PL, LA, LC, LL, LW, SD, and SL in which positive gains are desired, we used *η_nj_* = 100 and *φ_nj_* = 0. The correlation matrix of the original set of trait values (X*_ij_*) was maintained by the rescaled trait values (*r*X*_ij_*) in a two-way table in which each column with a range of 0–100 made a selection, i.e., increase.

In the second step, the factorial score of each test hybrid/genotype was estimated by performing EFA with rescaled values (*r*X*_ij_*) to group correlated traits into “*factors*”. By assuming *p* and *f* are the number of traits included and common factors retained through EFA, respectively, the scores were calculated as follows:


X=μ+Lf+ϵ

where *X* is a *p* × 1 vector of rescaled observations; *µ* is a *p* × 1 vector of standardized means; *L* is a *p* × *f* matrix of factorial loadings; *f* is a *p* × 1 vector of common factors; and *ϵ* is a *p* × 1 vector of residuals. Furthermore, the initial loadings were obtained by the traits having more than one eigenvalue that are acquired from the correlation matrix of *r*X*_ij_*. Then, final loadings were estimated by using *varimax* rotation criterion (Olivoto et al., 2019a) as given by:


$$F=Z{\left(A^{T}R^{-1}\right)}^{T}$$

where *F* is a *g* × *f* matrix with the factorial scores; *Z* is a *g* × *p* matrix with the standardized means (rescaled); *A* is a *p* × *f* matrix of canonical loadings; and *R* is a *p* × *p* correlation matrix between the traits. *g*, *f*, and *p* denote the number of test hybrids/genotypes (rows), factors retained (FA), and traits analyzed, respectively.

The ideotype (ID) was designed by assuming that it has the highest rescaled value, i.e., 100 for all the traits analyzed. Thus, the ID can be defined by 1 × *p* vector ID such that ID = [100, 100, …., 100]. The final scores for ID were also obtained according to the above formula. Finally, the MGIDI values were computed with the function *mgidi()* from the metan package. If *g* and *f* are the number of genotypes/rows and factors retained, respectively, the MGIDI for the *i*th genotype (MGIDIi) is calculated as follows:


$$MGIDI_{i}={\left[\sum_{j=1}^{f}{\left(\gamma_{ij}-\gamma_{j}\right)}^{2}\right]}^{0.5}$$

where *γ*_ij_ is the score of the *i*th genotype (row) in the *j*th factor (i =1, 2, …, g; j = 1, 2, …, f) and *γ_j_* is the jth score of the ideotype. The genotypes with the lowest MGIDI values, i.e., genotypes closer to the ID, exhibited the desired values for all the traits studied. The strengths and weaknesses of a genotype were represented by the proportion of the MGIDI index of the *i*th row/genotype explained by the *j*th factor (*ij*) estimated as follows:


$$\omega_{ij}=\frac{\sqrt{D_{ij}^{2}}}{\sum_{j=1}^{f}\sqrt{D_{ij}^{2}}}$$

where *D_ij_* is the distance between the *i*th genotype (row) and the ID for the *j*th factor. Low contributions of a factor specify that the traits within that factor are similar to the ideotype designed.

#### 2.5.4 Selection differential

The hybrid/genotypes were selected under different spacing trial environments through MGIDI values by assuming a selection intensity of ~15% and the selection differential in the percentage of population mean (*ΔS* %) was then computed for each trait as follows:


$$\Delta S\%=\frac{\left(X_{S}-X_{0}\right)}{X_{0}}\times 100$$

where *X_s_* and *X_0_* are the mean performance value of the selected hybrids and population (original population) mean, respectively.

### 2.6 Statistical software

All the statistical analyses were carried out on the RStudio, R version 4.1.3 (R Core Team, 2022) software with “metan” version v1.18.0 (Olivoto and Lúcio, 2020) and “ggplot2” version 3.3.4 (Wickham, 2016) packages. Functions such as *gamem_met()* for genotype analysis in multi-environments using mixed-effect or random-effect models, *gmd()* for extracting variance components, and *mgidi()* for the computation of MGIDI values were supplied. Pearson’s correlation coefficients were calculated using the means and BLUPs of genotypes across tested environments. The network plots of the pairwise correlation data frame were constructed by “corrr” package version 0.4.4 (Kuhn et al., 2022).

## 3 Results

### 3.1 Combined analysis of variance

The combined analysis of variance for each family and the joint family showed that most of 8 traits studied were significantly affected (P ≤ 0.001, P ≤ 0.01, and P ≤ 0.05) by genotype, environment, and GEI, except for SL which were not significantly influenced by genotype × environment (GEI) in both each family and the joint family (**Table 1**). Similarly, the environment effect was highly significant (P ≤ 0.001, P ≤ 0.01, and P ≤ 0.05) for all the evaluated traits except for 4 traits (LA, LL, LW, SD) in ‘1-XY’×‘N188’, two trait (H, PL) in ‘ZL-3’×‘N188’, another trait (H) in both ‘ZL-3’×‘N188’ and the joint family. Meanwhile, the effect of GEI was not significant (P ≤ 0.001, P ≤ 0.01, and P ≤ 0.05) for 3 traits (PL, LA, LL) in each family.

**Table 1:** Combined analysis of variance for 8 traits of the progeny of 3 families across the two spacing trial environments.

Family	Source	df	Mean Squares							
			H	PL	LA	LC	LL	LW	SD	SL
‘1-XY’×‘N139’	ENV	1	59.99***	5791.9***	34434***	11498***	207.9***	197.81***	432.5***	292.0***
	REP(ENV)	4	174.02***	1111.6***	5672***	283.5***	35.18***	38.58***	15.5ns	127.2***
	GEN	171	4.49***	158.0***	606***	76.3***	3.33***	3.04***	39.8***	15.7*
	GEN × ENV	129	2.80***	58.4ns	292ns	59.2ns	1.74ns	1.90*	40.1***	7.4ns
	Residuals	511	1.59	52.2	248	48.6	1.51	1.48	17.4	11.2
‘1-XY’×‘N188’	ENV	1	37.95***	817.1**	280ns	8318.3***	4.37ns	4.39ns	28.1ns	232.6***
	REP(ENV)	4	54.87***	425.7***	5575***	1028.8***	34.29***	29.45***	62.1***	13.7ns
	GEN	168	4.97***	549.7***	954***	96.4***	6.51***	5.23***	58.2***	16.7***
	GEN × ENV	130	2.26**	82.1ns	327ns	37.1ns	2.24ns	1.62ns	17.1ns	12.0ns
	Residuals	464	1.65	82.3	342	40.2	2.20	1.77	18.0	10.7
‘ZL-3’×‘N188’	ENV	1	6.17ns	5.0ns	1763**	3.77ns	7.44*	5.26*	79.1*	18.03ns
	REP(ENV)	4	85.09***	847.1***	1455***	179.06***	8.30***	8.72***	304.0***	52.31***
	GEN	231	3.54***	194.8***	488***	72.31***	3.90***	2.58***	35.2***	10.89*
	GEN × ENV	160	2.93***	55.8ns	299ns	38.66*	1.86ns	1.67*	18.2ns	10.07ns
	Residuals	654	1.91	49.4	263	29.67	1.56	1.28	19.7	8.95
Joint family	ENV	1	0.287ns	3247.5***	7639***	11823.6***	57.07***	58.18***	405.0***	1.73ns
	REP(ENV)	4	196.481***	993.3***	5904***	566***	35.54***	33.58***	148.6***	48.83**
	GEN	572	4.514***	393.7***	682***	85.7***	4.74***	3.61***	52.1***	15.14***
	GEN × ENV	421	2.914***	72.5*	373***	63.3***	2.32***	2.07***	24.8***	11.07ns
	Residuals	1637	2.013	62.8	296	40.7	1.82	1.57	19.0	10.74

*Significant at P ≤ 0.05; **Significant at P ≤ 0.01; ***Significant at P ≤ 0.001; ns, not significant.

ENV, environment (spacing trial); REP, replicate; GEN, genotypes; H, plant height (cm); PL, petiole length (cm); LA, leaf area (cm^2^); LC, leaf circumference (cm); LL, leaf length (cm); LW, leaf width (cm); SD, stomatal density; SL, stomatal length (μm).

### 3.2 Comparison between the BLUP means of 3 families over two spacing trials

**Figure 1:**
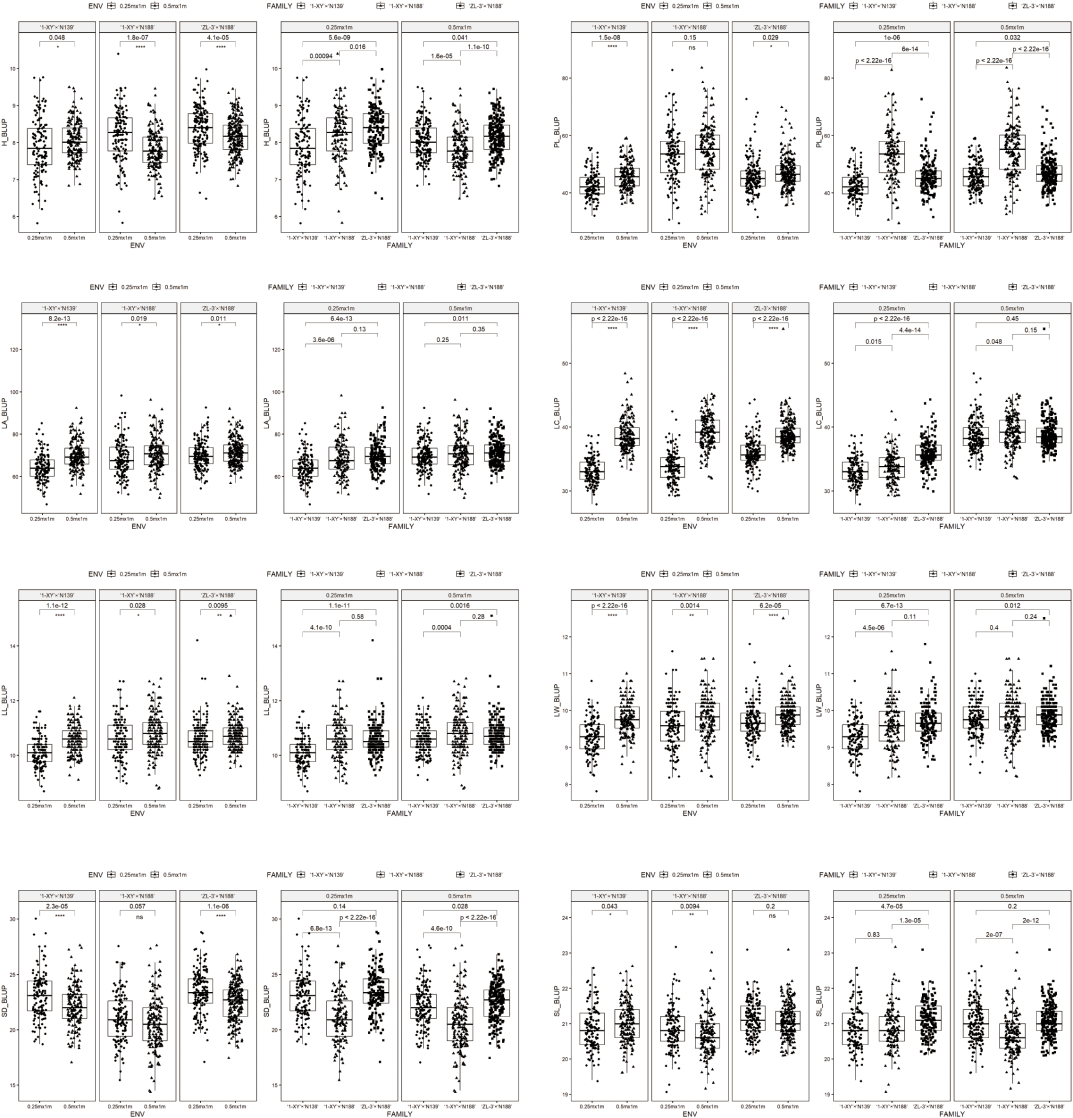
BLUP means of eight traits in the progenies from 3 families under two spacing trial environments including in 0.25 m × 1 m (E1) and 0.5 m × 1 m (E2). H, plant height (cm); PL, petiole length (cm); LA, leaf area (cm^2^); LC, leaf circumference (cm); LL, leaf length (cm); LW, leaf width (cm); SD, stomatal density; SL, stomatal length (μm); ENV, environments; GEN, genotypes.

The BLUP means of 8 traits including in growth, leaf morphology and stomatal characteristics were presented in **Figure 1** for the progeny of each family over two spacing trials (E1, E2). Significant differences (P ≤ 0.05) were observed between E1 and E2 for 5 traits (H, LC, LL, LA, LW) in each family. Moreover, the significant differences (P ≤ 0.05) between E1 and E2 were observed for one trait (SL) in both ‘1-XY’×‘N139’ and ‘1-XY’×‘N188’, and two traits (PL, SD) in both ‘1-XY’×‘N139’ and in ‘ZL-3’×‘N188’. The E1 trial (0.25 m x 1 m) revealed significant differences (P ≤ 0.05) for three traits (H, PL, LC) among the three families and four trait (LA, LL, LW) between ‘1-XY’×‘N139’ and ‘1-XY’×‘N188’ or ‘ZL-3’×‘N188’. Additionally, its results demonstrated significant differences (P ≤ 0.05) for one trait (SD) between ‘1-XY’×‘N188’ and ‘1-XY’×‘N139’ or ‘ZL-3’×‘N188’, as well as one trait (SL) between ‘ZL-3’×‘N188’ and ‘1-XY’×‘N139’ or ‘1-XY’×‘N188’. In the context of the E2 trial (0.5 m × 1 m), significant differences (P ≤ 0.05) were observed for three traits (H, PL, SD) among three families. The E2 trial also demonstrated statistically significant differences (P ≤ 0.05) for one trait (LL) between ‘1-XY’×‘N139’ and ‘1-XY’×‘N188’ or ‘ZL-3’×‘N188’and another trait (SL) between ‘1-XY’ × ‘N188’ and ‘1-XY’×‘N139’ or ‘ZL-3’×‘N188’, while two traits (LA, LW) between ‘1-XY’×‘N139’ and ‘ZL-3’×‘N188’ and another trait (LC) between ‘1-XY’×‘N139’ and ‘1-XY’×‘N188’.

The BLUP mean values of plant height (H) in ‘1-XY’×‘N139’ under E1 were found to be significantly (P ≤ 0.05) higher that under E2, while those in ‘1-XY’×‘N188’ and ‘ZL-3’×‘N188’ under E1 was significantly (P ≤ 0.05) lower than those under E2. The BLUP mean values of five traits (PL, LA, LC, LL, LW), exhibited reduction, among which three traits (LC, LL, LW) exhibited a significant (P ≤ 0.05) reduction, for three families under E1 in comparison to those observed under E2. Conversely, the BLUP mean values of one trait (SD) for three families exhibited increasing for three families and a significant (P ≤ 0.05) increasing in both ‘1-XY’×‘N139’ and ‘ZL-3’×‘N188’ under E1 in comparison to those observed under E2. The BLUP mean values of one trait (SL) under E1 were found to be significantly (P ≤ 0.05) lower in ‘1-XY’×‘N139’ and higher in ‘1-XY’×‘N188’ than those observed under E2. Furthermore, for the family, ‘1-XY’×‘N188’, the BLUP mean values of one trait (PL) under E1 were found to be significantly (P ≤ 0.05) lower than those observed under E2.

### 3.3 Variance components and genetic parameters

**Figure 2:**
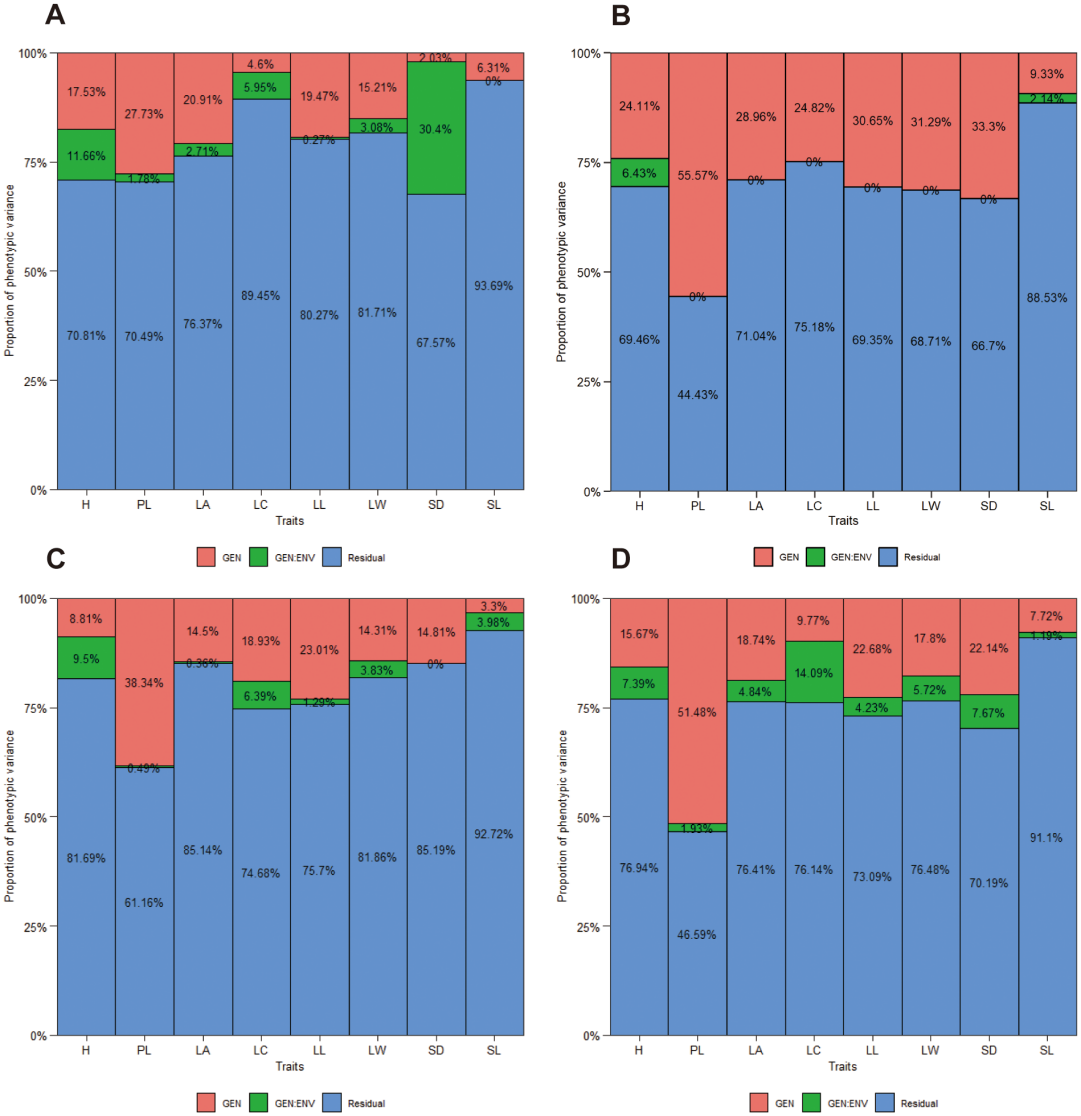
The proportion of phenotypic variance for 8 traits of three families, ‘1-XY’×’N139’ **(A)**, ‘1-XY’×’N188’ **(B)**, ‘ZL-3’×’N188’ **(C)** and the joint family **(D)** evaluated under two spacing trial environments. H, plant height (cm); PL, petiole length (cm); LA, leaf area (cm2); LC, leaf circumference (cm); LL, leaf length (cm); LW, leaf width (cm); SD, stomatal density; SL, stomatal length (μm).

The proportion of total variation explained by genotype, environment, and their interactions (GEI) for eight traits in each family and the joint family across spacing trial environments are shown in **Figures 2A–D**. The red column of genotypic variances were observed to be higher than the green column of genotype and environment interaction (GEI) variances for most of the traits in ach family (**Figures 2A–C**) and the joint family (Figure D), with exception of LC and SD in ‘1-XY’×‘N139’ (**Figure 2A**), H and SL in ‘ZL-3’×‘N188’ (**Figure 2C**) and LC in the joint family (**Figure 2D**). Additionally, PL exhibited the highest degree genetic variance than the other traits in each family and the joint family. Nevertheless, the proportions of total variation explained by genotype and GEI were found to be lower than those explained by the environment for all traits except for PL in ‘1-XY’×‘N188’ (**Figure 2B**) and the joint family (**Figure 2D**). Furthermore, the proportions of variation explained by GEI variance was approximately 0.00% for SL in ‘1-XY’×‘N139’ (**Figure 2A**), six traits (PL, LA, LC, LL, LW, SD) in ‘1-XY’×‘N188’ (**Figure 2B**) and one trait (SD) in ‘ZL-3’×‘N18’ (**Figure 2C**).

Genetic parameters for 8 traits of growth, leaf morphology and stomatal characteristics were shown in the **Table 2** for the poplar hybrids from each family and the joint family. Both broad-sense heritability and accuracy of hybrid selection were the highest for PL among the eight variables studied, both in each family and the joint family. For the heritability values of PL, the highest value was observed in the family ‘1-XY’×‘N188’ (0.556), while the lowest value was observed in ‘1-XY’×‘N139’ (0.277). The lowest heritability values (<0.1) were observed for SD (0.0203) in ‘1-XY’×‘N139’, SL (0.0933) in ‘1-XY’ × ‘N188’, H(0.0881) and SL (0.033) in ‘ZL-3’ × ‘N188’, LC(0.0977) and SL (0.0772) in joint family. The coefficient of determination of the interaction effects (GEIr^2^) showed the values of less than 0.304 for most traits in each family and the joint family exception for approximately equal values (0.00) for SL in ‘1-XY’×‘N139’, LA, LW, SD in ‘1-XY’ × ‘N188’, SD in ‘ZL-3’ × ‘N188’. The heritability on the mean basis (h^2^gm) showed values of great than half (>0.5) for most traits in each family and the joint family, except for SL (0.288), SD (0.0712), LC (0.205) and H (0.498) in ‘1-XY’×‘N139’, SL (0.371) in ‘1-XY’×‘N188’ and H (0.324), LW (0.479), SL (0.159) in ‘ZL-3’ × ‘N188’, H (0.487), LC (0.331) and SL (0.329) in the joint family. The accuracy of selection was high (>0.80) for most traits in each family and the joint family, except for some traits, including in the lowest (<0.50) for SD (0.267) and LC (0.452) in ‘1-XY’ × ‘N139’. The genotype–environment correlation (rge) displayed values of lower than half (<0.31) for all traits, indicating that the genotypic influence was an unsignificant contributor to the heritability of these traits in each family and the joint family. The genotypic CVs (CVg) were lower than that obtained from the residual CVs (CVr) for most traits in each family and the joint family, which indicates that the CVs (g/r) ratio was lower than 1, except for the trait, PL, in ‘1-XY’×‘N188’ and joint family, which were greater than 1.

**Table 2:** Genetic parameters for 10 traits of 3 families evaluated under two spacing trials.

Family	Genetic parameters	H	PL	LA	LC	LL	LW	SD	SL
‘1-XY’×‘N139’	Phenotypic variance	2.26	74	326	54.7	1.90	1.81	25.7	12
	Heritability	0.175	0.277	0.209	0.046	0.195	0.152	0.0203	0.0631
	GEIr^2^	0.117	0.0178	0.0271	0.0595	0.00265	0.0308	0.304	0.00
	h^2^gm	0.498	0.687	0.598	0.205	0.59	0.501	0.0712	0.288
	Accuracy	0.706	0.829	0.773	0.452	0.768	0.708	0.267	0.536
	rge	0.141	0.0247	0.0343	0.0624	0.00329	0.0363	0.31	0.00
	CVg	7.88	10.3	12.4	4.43	5.92	5.53	3.18	4.12
	CVr	15.8	16.4	23.7	19.5	12	12.8	18.4	15.9
	CV ratio	0.498	0.627	0.523	0.227	0.492	0.431	0.173	0.259
‘1-XY’×‘N188’	Phenotypic variance	2.45	184	476	51.7	3.18	2.54	26.8	12.2
	Heritability	0.241	0.556	0.29	0.248	0.307	0.313	0.333	0.0933
	GEIr^2^	0.0643	3.48E-09	0.00	3.48E-16	5.81E-10	0.00	0.00	0.0214
	h^2^gm	0.62	0.882	0.71	0.664	0.726	0.732	0.75	0.371
	Accuracy	0.787	0.939	0.843	0.815	0.852	0.856	0.866	0.609
	rge	0.0847	7.84E-09	0.00	4.63E-16	8.37E-10	0.00	0.00	0.0236
	CVg	9.87	18.2	16.7	9.60	9.10	9.13	14.9	5.27
	CVr	16.8	16.2	26.1	16.7	13.7	13.5	21.1	16.2
	CV ratio	0.589	1.12	0.639	0.575	0.665	0.675	0.707	0.325
‘ZL-3’×‘N188’	Phenotypic variance	2.42	83.1	315	41	2.13	1.62	22.9	9.52
	Heritability	0.0881	0.383	0.145	0.189	0.23	0.143	0.148	0.033
	GEIr^2^	0.095	0.00494	0.0036	0.0639	0.0129	0.0383	0.00	0.0398
	h^2^gm	0.324	0.786	0.502	0.548	0.634	0.479	0.51	0.159
	Accuracy	0.569	0.887	0.709	0.74	0.796	0.692	0.714	0.399
	rge	0.104	0.00801	0.00421	0.0788	0.0168	0.0447	0.00	0.0411
	CVg	5.52	12.2	9.41	7.26	6.53	4.88	7.92	2.62
	CVr	16.8	15.4	22.8	14.4	11.8	11.7	19	13.9
	CV ratio	0.328	0.792	0.413	0.503	0.551	0.418	0.417	0.189
Joint family	Phenotypic variance	2.67	136	393	54.4	2.55	2.10	27.1	11.7
	Heritability	0.157	0.515	0.187	0.0977	0.227	0.178	0.221	0.0772
	GEIr^2^	0.0739	0.0193	0.0484	0.141	0.0423	0.0572	0.0767	0.0119
	h^2^gm	0.487	0.855	0.553	0.331	0.613	0.533	0.588	0.329
	Accuracy	0.698	0.925	0.744	0.575	0.783	0.73	0.767	0.573
	rge	0.0876	0.0398	0.0596	0.156	0.0547	0.0696	0.0985	0.0128
	CVg	8.01	17.3	12.3	6.18	7.15	6.29	11.1	4.54
	CVr	17.8	16.5	24.8	17.3	12.8	13	19.7	15.6
	CV ratio	0.451	1.05	0.495	0.358	0.557	0.482	0.562	0.291

H, plant height (cm); PL, petiole length (cm); LA, leaf area (cm2); LC, leaf circumference (cm); LL, leaf length (cm); LW, leaf width (cm); LR, leaf round; SD, stomatal density; SN, stomatal number; SL, stomatal length (μm).

### 3.4 Correlations and cluster analysis

**Figure 3:**
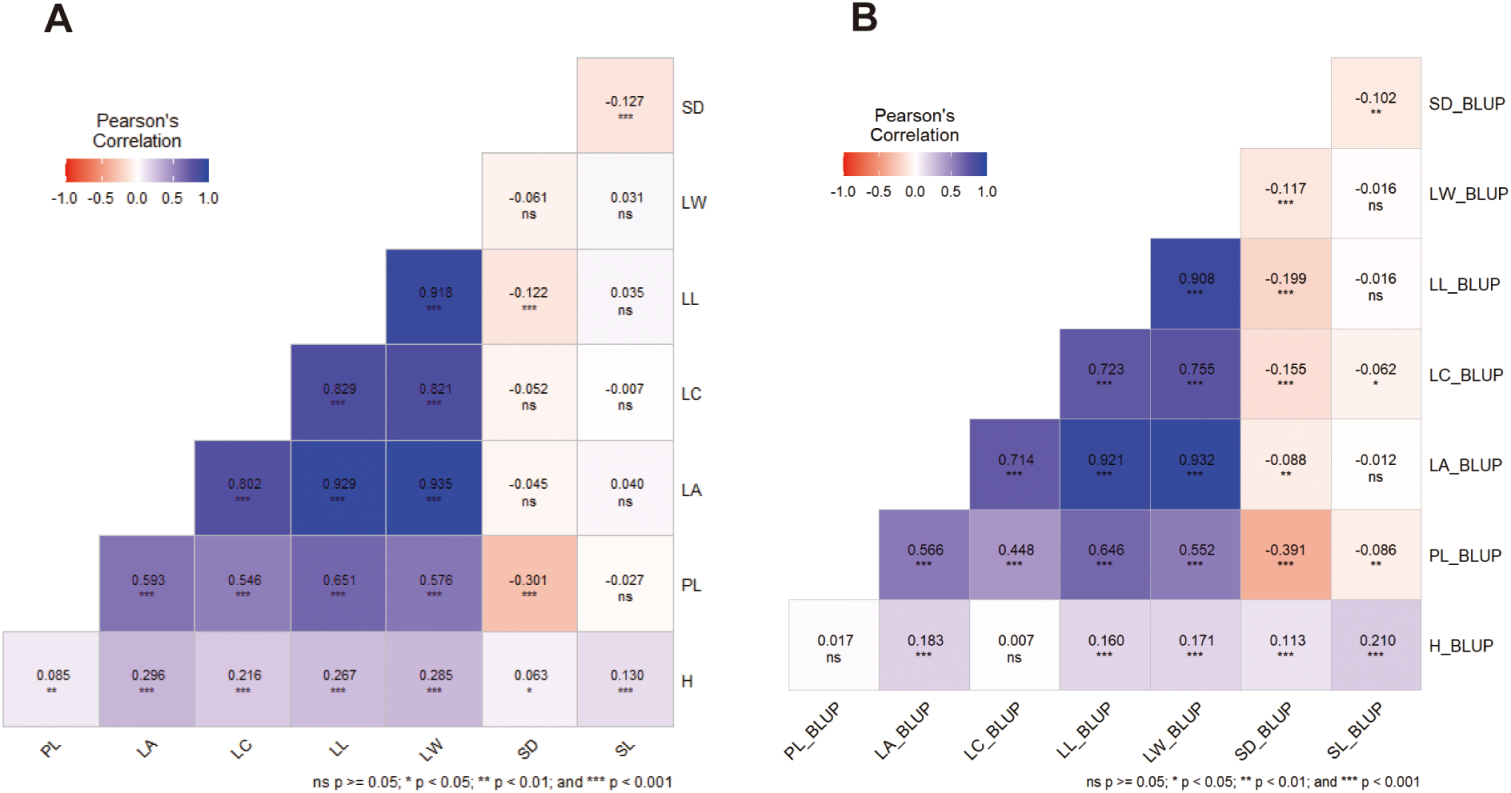
Pairwise correlation (Pearson’s coefficients) using the means **(A)** and BLUPs **(B)** of 8 traits of 3 families grown across the 2 distinct spacing trials. H, plant height (cm); PL, petiole length (cm); LA, leaf area (cm2); LC, leaf circumference (cm); LL, leaf length (cm); LW, leaf width (cm); SD, stomatal density; SL, stomatal length (μm).

Pairwise correlation based on Pearson’s coefficients was used to examine the correlations between the means (**Figure 3A**) and BLUPs (**Figure 3B**) of 8 traits. Significant positive correlations (P ≤ 0.001, P ≤ 0.01, and P ≤ 0.05) were found between growth (H) and leaf morphology (PL, LA, LC, LL, LW) or stomatal characteristics (SD, SN) on both means and BLUPS exception for that between BLUPs of H and LL. Notably, strong negative correlations (P ≤ 0.01) of means or BLUPs were showed pairwise among two stomatal characteristics (SD, SL) or five leaf morphological traits (PL, LA, LC, LL, LW). Negative correlations were showed between BLUPs of leaf morphology (PL, LA, LC, LL, LW) and stomatal characteristics (SD, SN). Conversely, means of SD showed significant negative correlations (p ≤ 0.001) with PL (r=-0.304), LL (r=-0.122) and SL (r=-0.127). The results were shown that for the correlations between leaf morphology and stomatal characteristics, SD:(LA, LC, LW) and SL:(PL, LA, LC, LL, LW) showed no significance on means (**Figure 3A**), as well as these of H:PL (LC) and SL:(LA, LL, LW) on BLUPs (**Figure 3B**). Furthermore, the correlation coefficients between the BLUPs of the traits were lower than those between the means of phenotypic data for the trait studies.

**Figure 4:**
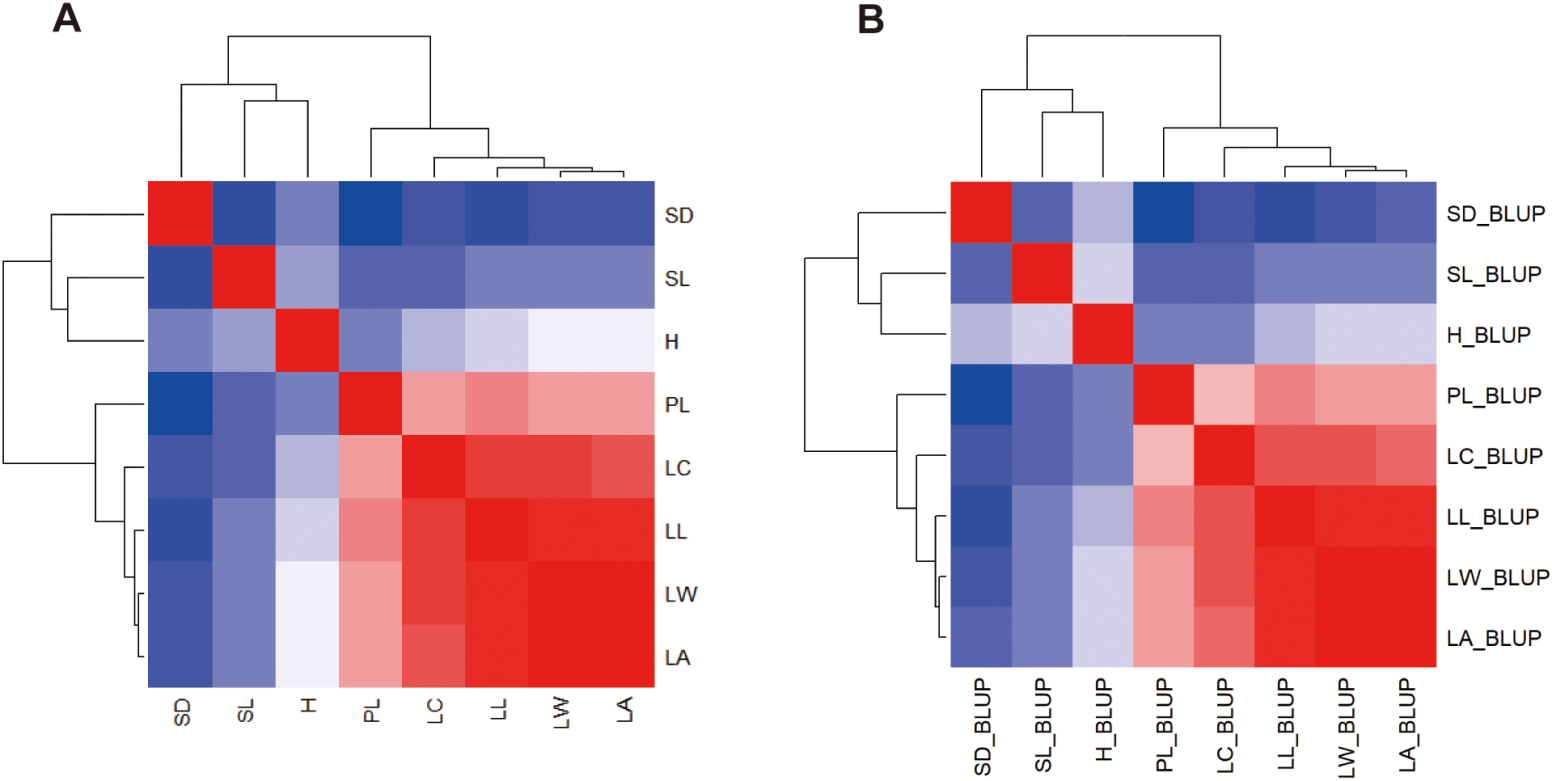
Heatmap clustering using the means **(A)** and BLUPs **(B)** of 8 traits in the progenies of 3 families grown across two distinct spacing trials. The intensity of color in each figure corresponds to the value of each estimate.

Using heatmap cluster analysis for the 8 traits studied, two distinct clusters were observed in the plots of both the means (**Figure 4A**) and the BLUPs (**Figure 4B**). The first cluster comprised the five leaf morphological characters with a clear clustering among LA, LW, LL, LC and PL. The second cluster consisted of growth (H) and the two stomatal characteristics (SN, SL) with a clear clustering.

### 3.5 Loadings and factor description for MGIDI

According to the final loadings obtained from PCA followed by EFA, two factors (FAs with more than 1 eigenvalue) accounting for 68.8% of the total variability were retained for the progeny of ‘ZL-3’×‘N188’, whereas three factors accounting for 78.6%, 86.3% and 82.0% of the total variability were retained for ‘1-XY’×‘N139’, ‘1-XY’×‘N188’ and the joint family, respectively (**Table 3**). For ‘ZL-3’×‘N188’, growth (H) and five leaf morphological characters (PL, LA, LC, LL, LW) were included in FA1; two stomatal characteristics (SD, SN) were included in FA2. Among the three factors retained for both ‘1-XY’×‘N139’ and ‘1-XY’×‘N188’, FA1 included five leaf morphological characters (PL, LA, LC, LL, LW); FA2 included two stomatal characteristics (SD, SN); FA3 included growth (H). Similarly, for the joint family, FA1 included five leaf morphological characters (PL, LA, LC, LL, LW); FA2 included SD; FA3 included H and SL.

**Table 3:** Eigenvalues, explained variance, cumulative variance, and final loadings of factors retained after superposition by EFA and selection gains based on MGIDI values.

‘1-XY’×’N139’											
Traits	FA1	FA2	FA3	Communality	Uniquenesses	VAR	Factor	Xo	Xs	SD	SD%
H	-0.0402	0.0479	0.893	0.801	0.199	PL	FA1	44	45.7	1.68	3.81
PL	-0.759	-0.0284	-0.00212	0.577	0.423	LA	FA1	66.5	71.1	4.57	6.87
LA	-0.975	0.0185	0.0262	0.951	0.0489	LC	FA1	35.8	36.2	0.412	1.15
LC	-0.885	0.0429	-0.045	0.786	0.214	LL	FA1	10.3	10.6	0.332	3.23
LL	-0.964	-0.00822	0.0123	0.929	0.0706	LW	FA1	9.50	9.76	0.258	2.72
LW	-0.952	0.00619	0.0237	0.908	0.0924	SD	FA2	22.7	22.8	0.0734	0.324
SD	0.0627	0.805	0.253	0.715	0.285	SL	FA2	21.1	21.1	0.066	0.313
SL	0.0931	-0.655	0.426	0.619	0.381	H	FA3	7.99	8.56	0.569	7.13
Eigenvalues	4.16	1.10	1.02								
Variance (%)	52	13.8	12.8								
Cum.variance(%)	52	65.8	78.6								
‘1-XY’×’N188’											
Traits	FA1	FA2	FA3	Communality	Uniquenesses	VAR	Factor	Xo	Xs	SD	SD%
H	-0.163	-0.0788	-0.897	0.837	0.163	PL	FA1	55.6	57.8	2.13	3.83
PL	-0.78	0.299	0.164	0.725	0.275	LA	FA1	70.4	77.6	7.26	10.3
LA	-0.955	-0.0201	-0.146	0.934	0.0661	LC	FA1	37.3	39	1.69	4.53
LC	-0.952	0.0359	-0.0528	0.91	0.0903	LL	FA1	10.9	11.3	0.488	4.49
LL	-0.966	0.075	-0.0809	0.945	0.0547	LW	FA1	9.76	10.3	0.507	5.19
LW	-0.951	-0.00711	-0.121	0.92	0.0805	SD	FA2	20	20.8	0.777	3.89
SD	0.182	-0.877	-0.17	0.831	0.169	SL	FA2	20.2	20.5	0.291	1.44
SL	0.109	0.682	-0.568	0.8	0.2	H	FA3	7.78	8.58	0.806	10.4
Eigenvalues	4.42	1.34	1.15								
Variance (%)	55.2	16.7	14.3								
Cum.variance(%)	55.2	71.9	86.3								
‘ZL-3’×’N188’											
Traits	FA1	FA2	FA3	Communality	Uniquenesses	VAR	Factor	Xo	Xs	SD	SD%
H	-0.372	0.122	0.154	0.846	-0.372	H	FA1	8.36	8.47	0.108	1.30
PL	-0.685	-0.255	0.535	0.465	-0.685	PL	FA1	46.2	52.5	6.32	13.7
LA	-0.913	0.0651	0.838	0.162	-0.913	LA	FA1	71.9	78.8	6.97	9.70
LC	-0.948	-0.0188	0.899	0.101	-0.948	LC	FA1	38.4	41.4	3.03	7.90
LL	-0.95	-0.0541	0.906	0.0937	-0.95	LL	FA1	10.7	11.5	0.812	7.56
LW	-0.935	0.0364	0.876	0.124	-0.935	LW	FA1	9.88	10.3	0.46	4.65
SD	0.0513	0.821	0.677	0.323	0.0513	SD	FA2	23.2	22.9	-0.353	-1.52
SL	0.0656	-0.798	–	0.64	0.36	SL	FA2	21.4	21.4	-0.0131	-0.0612
Eigenvalues	4.13	1.38	–								
Variance (%)	51.6	17.2	–								
Cum.variance(%)	54.5	68.8	–								
Joint family											
Traits	FA1	FA2	FA3	Communality	Uniquenesses	VAR	Factor	Xo	Xs	SD	SD%
H	0.227	0.412	-0.626	0.614	0.386	PL	FA1	48.3	52	3.70	7.67
PL	0.657	-0.488	0.162	0.695	0.305	LA	FA1	69.8	77.6	7.84	11.2
LA	0.955	0.0277	-0.0602	0.916	0.0844	LC	FA1	37.3	38.7	1.45	3.89
LC	0.93	-0.00504	-0.0328	0.866	0.134	LL	FA1	10.6	11.3	0.682	6.41
LL	0.961	-0.108	-0.0317	0.937	0.0633	LW	FA1	9.73	10.2	0.503	5.17
LW	0.952	0.0132	-0.0597	0.909	0.0908	SD	FA2	22.1	22.4	0.229	1.03
SD	-0.0645	0.901	0.0854	0.823	0.177	H	FA3	8.08	8.43	0.353	4.37
SL	-0.096	-0.227	-0.86	0.8	0.2	SL	FA3	21	21.2	0.239	1.14
Eigenvalues	4.15	1.29	1.12								
Variance (%)	51.8	16.2	14.0								
Cum.variance(%)	51.8	68.0	82.0								

FA1, factor 1; FA2, factor 2; FA3, factor3; H, plant height (cm); PL, petiole length (cm); LA, leaf area (cm2); LC, leaf circumference (cm); LL, leaf length (cm); LW, leaf width (cm); SD, stomatal density; SL, stomatal length (μm).

### 3.6 Multi-trait genotype–ideotype distance index and selection gains

**Figure 5:**
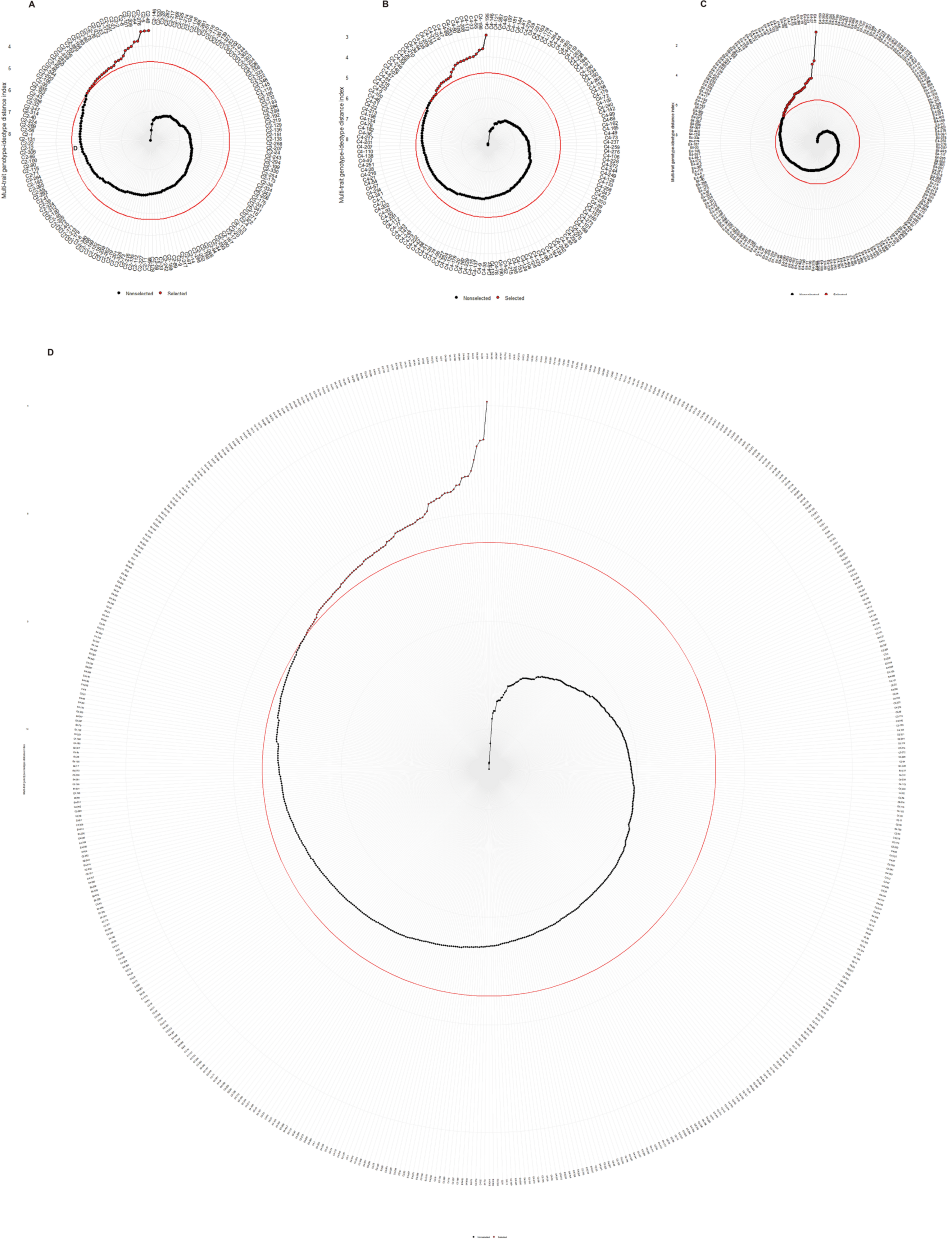
Genotype ranking in ascending order for the MGIDI index tested for the progeny genotypes of family,’1-XY’×’N139’ **(A)**, ‘1-XY’×’N188’ **(B)**, ‘ZL-3’×’N188’ **(C)**, and the joint family **(D)**. The selected genotypes were shown in red color and the red colored circle represents the cut-point according to the selection pressure (~15%).

The best performing progeny genotypes from each family and the joint family were determined using the factor analysis, in which the traits were grouped into two or three factors (**Table 3**). The MGIDI index was calculated to identify the best performing progeny genotypes of each family and the joint family when considering all of 8 traits studied. The MGIDI analysis, by assuming a selection index of 15%, identified 26, 25, 35, and 86 hybrid genotypes for best performing in three families, ‘1-XY’×‘N139’ (**Figure 5A**), ‘1-XY’×‘N188’ (**Figure 5B**), ‘ZL-3’×‘N188’ (**Figure 5C**), and the joint family (**Figure 5D**), respectively. The selected genotypes displayed the potential for simultaneous improvement of the studied traits in a poplar improvement program. As for selection gains based on the MGIDI index (**Table 3**), the traits PL showed the highest genetic gains (3.81% - 13.7%), while SD showed the lowest genetic gains (-1.52% - 3.89%) for all the studied traits in all the families and the joint family (**Table 3**). In addition, growth trait (H) showed the genetic gain in range of from 1.3% in ‘ZL-3’×‘N188’ to 7.67% in the joint family.

### 3.7 The strengths and weaknesses view of selected hybrid progenies

**Figure 6:**
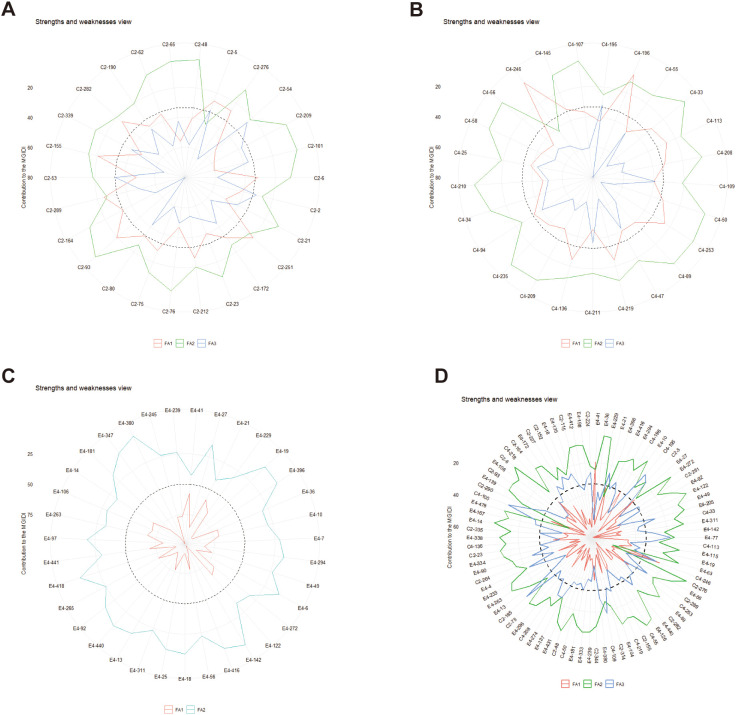
The strengths and weaknesses view of the selected progeny genotypes in the families, namely, C2, ‘1-XY’×’N139’ **(A)**; C4, ‘1-XY’×’N188’ **(B)**; E4, ‘ZL-3’×’N188’ **(C)**; and the joint family **(D)** was shown as the proportion of each factor on the computed MGIDI values. The smallest the proportion explained by a factor (closer to the external edge), the closer the traits within that factor are to the ideotype. The dashed line shows the theoretical value if all the factors had contributed equally.

The radar plots (**Figures 6A–D**) depicts the strengths and weaknesses of the selected progeny genotypes of each family and the joint family over two spacing trials. For each selected genotypes, the contribution of each factor towards the MGIDI is ranked from the most contributing factor (close to plot center) to the least contributing factor (away from the plot center). Smaller proportions explained by a factor that is placed closer to the external edge indicate that the trait within that factor is more similar to the ideotype.

A view on strengths and weaknesses across all families revealed that the performance of the selected genotypes, viz., C2-155 of ‘1-XY’×‘N139’ (**Figure 6A**), C4-246 of ‘1-XY’×‘N188’ (**Figure 6B**), E4-41 of ‘ZL-3’×‘N188’ (**Figure 6C**), and E4-41, C4-246, 218 of the joint family (**Figure 6D**) showed strengths related to factor FA1 that holds leaf morphological traits (PL, LA, LC, LL, LW), whereas genotypes C2-93, 48 of ‘1-XY’×‘N139’ (**Figure 6A**), C4-210, 235, 56, 107 of ‘1-XY’×‘N188’ (**Figure 6B**), and C2-6, C4-55, E4-14 of the joint family (**Figure 6D**) showed strengths related to FA2 with stomata density (SD and SN). Concerning FA3, genotypes C2-54 of ‘1-XY’×‘N139’ (**Figure 6A**), C4-195 of ‘1-XY’×‘N188’ (**Figure 6B**), and C2-185, C4-100, 185 of the joint family performed well (**Figure 6D**). For the family ‘ZL-3’×‘N188’ (**Figure 6C**), most of the selected hybrids contributed more towards MGIDI through FA2 including stomata characters (SD, SL) except E4-380, 27, and 142 (**Figure 6C**). Similarly for the joint family (**Figure 6D**), most of the selected genotypes showed strengths related to F2 (SD, SL) and weaknesses related to FA1 (PL, LA, LC, LL, LW) and FA3 (H).

## 4 Discussion

### 4.1 Variation, genetic parameters and genotype by spacing interaction for straits

The principal objective of poplar breeding programme is to develop new genotypes or varieties that are resistant to biotic and abiotic stresses with improved genetic gains, performance, stability, and adaptability in comparison to traditional cultivars (Biselli et al., 2022; Bisoffi and Gullberg, 1996). The accurate assessment of numerous testing genotypes in traditional genetic trials following the initial growing season is crucial for the identification of a small number of genotypes with the potential for superior growth and other desired characteristics (Ren et al., 2020; Yáñez et al., 2019; Gebauer et al., 2016; Sixto et al., 2011). In this study, we evaluated the diversity and consistency of growth traits among a large set of progeny genotypes representing a diverse range of parental backgrounds. These hybrids were cultivated under two distinct planting spacing trial environments. Given the high cost and timing involved in the selection of hybrids for SRC regimes, we also focused on the potential for varietal selection after the first growing season, as previously mentioned by Gudynaitė-Franckevičienė and Pliūra (2022); Yáñez et al. (2019) and Sixto et al. (2011). Their results supported the hypothesis that genetic parameters measured in the first growing season are essential indicators of the temporal performance of poplar growth under SRC. The results demonstrated that the progeny genotypes of *P. simonigra* exhibited significant differences in growth, leaf and stomatal morphological traits in three families across two distinct plant spacing trials, namely 0.5 m × 1 m (E1) and 0.25 m × 1 m (E2). These differences were attributed to the varying phenotypic plasticity of the genotypes in response to the disparate environmental conditions of the plant spacing trials. These findings indicate that the traits exhibit considerable genetic potential of the progeny among the tested three families, and that the selection of excellent genotypes and families is viable possibility. The examined traits were also found to exhibit significant effects based on the spacing trial environments, indicating that poplar hybrids display considerable phenotypic plasticity. These findings have also been corroborated by a substantial body of prior research (Gardiner et al., 2024; Gudynaitė-Franckevičienė and Pliūra, 2022 and Gudynaitė-Franckevičienė V., and Pliūra, A. (2021); Ghezehei et al., 2021; Ren et al., 2020; Yáñez et al., 2019; Nelson et al., 2018; Sixto et al., 2011).

More than 50% of the phenotypic variance observed in all studied traits, with the exception of PL in three families and the joint family, can be attributed to environmental effects and GEI interactions. This has the effect of reducing heritability. The significant genotype main effect and G×E interactions suggested that the progeny genotypes of the three families exhibited different responses in the distinct plant spacing trials. Such plant spacing as environmental interaction has a significant impact on complex quantitative traits with several contributing factors such traits as growth and productivity, which can be measured during the first growing season and served as stable and additional selection criteria (Ghezehei et al., 2021; Tsarev et al., 2021; Yáñez et al., 2019; Sixto et al., 2011). The differences between E1 and E2 led to differences in light and soil resources of the forest land during the initial growing season (Gardiner et al., 2024; Ding et al., 2022; Yáñez et al., 2019).

When poplar selection and breeding, it is crucial to ascertain which environmental factors are the primary determinants of the G×E. Further multi-environmental experiments can be carried out to integrate the mutual effects of different environmental factors and to assess the adaptability and genetic stability of poplar in a more comprehensive way. This knowledge provides a crucial reference point for the division of suitable planting environments, thereby facilitating the improvement of poplar genotypes or varieties. It is essential to analyze mean performance, genetic variation, parameters and determine stability and GEI in order to facilitate effective breeding and adaptability in a wide range of environmental conditions (Gudynaitė-Franckevičienė and Pliūra, 2022; Ghezehei et al., 2021; Li, 2021; Niemczyk and Thomas, 2020; Ding et al., 2020; Ren et al., 2020; Yáñez et al., 2019; Sixto et al., 2011).

In the context of a specific genotype set, it can be observed that an increase in site heterogeneity is associated with a corresponding rise in G×E amplitude. Furthermore, the differing responses to environmental changes among genotypes represent the internal driving force generated by the G×E (Li et al., 2017). Consequently, the analysis of the G × E enables the estimation of the actual genetic parameters of environmental quantitative traits in a multitude of genetic testing trials. Furthermore, these analyses provide a foundation for the selection of genotypes with superior phenotypes and stable genetics, as well as the optimization of environmental evaluations. The objective of enhancing production and efficiency in artificial timber forests can be achieved by selecting the most suitable genotypes for the given environment (Yuan et al., 2022; Li, 2021). The results of this study, however, have further revealed that the environmental coefficients of variance of the tested traits were greater than the genetic coefficients of variance. Furthermore, the spacing trial factors had a substantial contribution rate to the growth, leaf morphology and stomata characteristics of the progeny genotypes of three families. These findings indicated that the environmental factor of the spacing trials had a more pronounced impact on the observer variances in the corresponding traits than the G factor. The weak heritability coefficient indicated a strong interaction between the genotype and the environmental factor of the spacing trial. This phenomenon is also consistent with previous research on aspen (Ding et al., 2020), poplar hybrids and clones (Gudynaitė-Franckevičienė and Pliūra, 2022; 2021; Nelson et al., 2018; Sixto et al., 2011).

### 4.2 Selection of genotypes via multi-trait index - MGIDI

Although the economic benefits of poplar plantation are dependent on their growth traits, the contributions of their leaf and stomata morphology to their productivity cannot be ignored (Otis-Prud’homme et al., 2023; Grady et al., 2013; Verlinden et al., 2013; Al Afas et al., 2006; 2007). Marron and Ceulemans (2006); Marron et al. (2007 and 2005) and Gebauer et al. (2016) found that petiole and leaf traits of poplar were closely related to growth traits became an adaptive trait and early indicator of biomass yield. Modern techniques such as genetic linkage analysis could be combined to further reveal the genetic basis and regulatory network of the traits and genetic basis of these traits can be further explored in future studies. In this study a strong correlation was observed between the means and BLUPs of height and leaf traits, respectively. Therefore, direct selection for these traits may not be advantageous due to their low heritability, which limits overall genetic gain. However, in cases where there are high magnitudes of correlation and heritability, it is possible that indirect measurement and selection based on initial characteristics of plant growth and development may be efficient in identifying desirable genotypes, thereby reducing the selection cycle (Niemczyk and Thomas, 2020). Addressing multicollinearity issues is crucial to avoid bias in genetic parameter estimates during selection, especially when using conventional indices like Smith (1936). Therefore, it is crucial to extend conventional selection methods to encompass multiple traits in order to maximize genetic gains, taking into account the correlated genetic relationships among traits, as well as the presence of pleiotropy and genetic linkage (Du et al., 2022). Additionally, assigning appropriate economic weights to agronomically important traits remains a challenge, often leading to suboptimal gains per generation (Cerón-Rojas et al., 2006). Establishing correlations between stability and agronomic performance for primary traits poses a significant challenge in poplar breeding.

Improvements in morphological heritability are thus also considered an important component in the poplar breeding. Recommendations based on multi-trait analysis are considered more reliable than single-trait analysis, particularly when the evaluated traits are highly correlated. The multi-trait genotype-ideotype distance (MGIDI) index was used to select the progeny genotypes taking into account all the measured traits (Olivoto and Nardino, 2021; Olivoto et al., 2019b). The process involves scaling the trait using BLUP for genotype mean performance, calculating the factor analysis, and determining the distance of each genotype from the ideotype (Rocha et al., 2018). By using a two-way table as input data and allowing rows to be ranked based on desired outcomes in the columns, it provided an effective means of evaluating the strengths and weaknesses of the selected genotypes. Furthermore, with the ability to evaluate multiple dependent traits, it proves to be a valuable tool in such evaluations. The predicted genetic gain for effective traits in the MGIDI index is shown in **Table 3**. The results indicated a higher percentage of genetic gain for key measured traits, such as height, leaf shape and stomatal density and size. The selected traits with the highest genetic gains were LA (6.87% - 11.2%) and PL (3.81% - 13.7%). The trait of plant height showed an increased selection gain of 1.3% - 10.4%. SD and SL of the stomata characteristics showed selection gains of the lowest in family ‘1-XY’×‘N139’ (0.324% and 0.313%) and ‘ZL-3’×‘N188’ (-1.52% and -0.0612%), and the highest in family ‘1-XY’×‘N188’ (3.89% and 1.44%).

In recent years, as breeding programs have advanced and the production system has become more modernized, there has been an increasing demand for simultaneous improvement of multiple traits through multivariate approaches, driven by the evolving requirements of the poplar industry. The goal of poplar breeding has thus changed from the selection of single traits to the comprehensive selection of multiple traits. However, owing to the inconsistent performances of the growth and morphological traits, it is often challenging to simultaneously improve the two trait types (Adler et al., 2021; Pilipović et al., 2019; Gebauer et al., 2016; Grady et al., 2013). In the present study, multiple trait selection index was used to select the progeny genotypes with excellent comprehensive performances. These selected hybrids of three families not only maintained their fast-growing characteristics but their morphological traits also performed well, such that their breeding efficiencies were in line with expectations. Considering that the tested genotypes were one years old and provided a valuable addition to the clonal selection for rapid juvenile growth, especially where very rotations are desired (no more than 3 years). In any case, determining clonal progeny stability in terms of growth is of great use not only when deciding on the genotypes during the vegetative propagation phase to be used in plantations but also when developing breeding programs (Gudynaitė-Franckevičienė and Pliūra, 2022; 2021; Ren et al., 2020; Yáñez et al., 2019; Sixto et al., 2011). These findings provide a theoretical basis for the efficient cultivation of poplar hybrids in commercial timber plantation in North China.

## 5 Conclusion

Our experimental findings recommended that MGIDI can be used for the effective selection of superior hybrids/genotypes by considering multiple traits and helping plant breeders make better strategic decisions. The results showed that 26, 25, 35, and 86 selected hybrid genotypes of the three families and the joint family, respectively, exhibited the desired genetic gains, including positive gains for plant height and leaf morphological characters. The tested progeny genotypes of three families provided a valuable addition to the hybrid selection for rapid juvenile growth.

## Data Availability

The original contributions presented in the study are included in the article/supplementary material. Further inquiries can be directed to the corresponding author.
